# Joint EANM/SNMMI guideline on radiomics in nuclear medicine: Jointly supported by the EANM Physics Committee and the SNMMI Physics, Instrumentation and Data Sciences Council

**DOI:** 10.1007/s00259-022-06001-6

**Published:** 2022-11-03

**Authors:** M. Hatt, A. K. Krizsan, A. Rahmim, T. J. Bradshaw, P. F. Costa, A. Forgacs, R. Seifert, A. Zwanenburg, I. El Naqa, P. E. Kinahan, F. Tixier, A. K. Jha, D. Visvikis

**Affiliations:** 1 LaTIM, INSERM, UMR 1101, Univ Brest, Brest, France; 2 ScanoMed Ltd., Debrecen, Hungary; 3 Departments of Radiology and Physics, University of British Columbia, Vancouver, BC Canada; 4 Department of Radiology, University of Wisconsin, Madison, WI USA; 5 Department of Nuclear Medicine, West German Cancer Center, University of Duisburg-Essen and German Cancer Consortium (DKTK)-University Hospital Essen, Essen, Germany; 6 Department of Nuclear Medicine, Münster University Hospital, Münster, Germany; 7 OncoRay—National Center for Radiation Research in Oncology, Faculty of Medicine and University Hospital Carl Gustav Carus, Technische Universität Dresden, Helmholtz-Zentrum Dresden-Rossendorf, Dresden, Germany; 8 National Center for Tumor Diseases (NCT/UCC), Dresden, Germany; 9 German Cancer Research Center (DKFZ), Heidelberg, Germany; 10 Department of Machine Learning, Moffitt Cancer Center, Tampa, FL 33626 USA; 11 Imaging Research Laboratory, PET/CT Physics, Department of Radiology, UW Medical Center, University of Washington, Seattle, WA USA; 12 McKelvey School of Engineering and Mallinckrodt Institute of Radiology, Washington University in St. Louis, Saint Louis, MO USA

**Keywords:** Radiomics, Machine learning, Deep learning, Nuclear medicine, Recommendations

## Abstract

**Purpose:**

The purpose of this guideline is to provide comprehensive information on best practices for robust radiomics analyses for both hand-crafted and deep learning-based approaches.

**Methods:**

In a cooperative effort between the EANM and SNMMI, we agreed upon current best practices and recommendations for relevant aspects of radiomics analyses, including study design, quality assurance, data collection, impact of acquisition and reconstruction, detection and segmentation, feature standardization and implementation, as well as appropriate modelling schemes, model evaluation, and interpretation. We also offer an outlook for future perspectives.

**Conclusion:**

Radiomics is a very quickly evolving field of research. The present guideline focused on established findings as well as recommendations based on the state of the art. Though this guideline recognizes both hand-crafted and deep learning-based radiomics approaches, it primarily focuses on the former as this field is more mature. This guideline will be updated once more studies and results have contributed to improved consensus regarding the application of deep learning methods for radiomics. Although methodological recommendations in the present document are valid for most medical image modalities, we focus here on nuclear medicine, and specific recommendations when necessary are made for PET/CT, PET/MR, and quantitative SPECT.

**Supplementary Information:**

The online version contains supplementary material available at 10.1007/s00259-022-06001-6.

## Preamble

The Society of Nuclear Medicine and Molecular Imaging (SNMMI) is an international scientific and professional organization founded in 1954 to promote the science, technology, and practical application of nuclear medicine. The European Association of Nuclear Medicine (EANM) is a professional non-profit medical association that facilitates communication worldwide between individuals pursuing clinical and research excellence in nuclear medicine. The EANM was founded in 1985. SNMMI and EANM members are physicians, technologists, and scientists specializing in the research and practice of nuclear medicine.

The SNMMI and EANM will periodically define new guidelines for nuclear medicine practice to help advance the science of nuclear medicine and to improve the quality of service to patients throughout the world. Existing practice guidelines will be reviewed for revision or renewal, as appropriate, on their fifth anniversary or sooner, if indicated.

Each practice guideline, representing a policy statement by the SNMMI/EANM, has undergone a thorough consensus process in which it has been subjected to extensive review. The SNMMI and EANM recognize that the safe and effective use of diagnostic nuclear medicine imaging requires specific training, skills, and techniques, as described in each document. Reproduction or modification of the published practice guideline by those entities not providing these services is not authorized.

These guidelines are an educational tool designed to assist practitioners in providing appropriate care for patients. They are not inflexible rules or requirements of practice and are not intended, nor should they be used, to establish a legal standard of care. For these reasons and those set forth below, both the SNMMI and the EANM caution against the use of these guidelines in litigation in which the clinical decisions of a practitioner are called into question.

The ultimate judgment regarding the propriety of any specific procedure or course of action must be made by the physician or medical physicist in light of all the circumstances presented. Thus, there is no implication that an approach differing from the guidelines, standing alone, is below the standard of care. To the contrary, a conscientious practitioner may responsibly adopt a course of action different from that set forth in the guidelines when, in the reasonable judgment of the practitioner, such course of action is indicated by the condition of the patient, limitations of available resources, or advances in knowledge or technology subsequent to publication of the guidelines.

The practice of medicine includes both the art and the science of the prevention, diagnosis, alleviation, and treatment of disease. The variety and complexity of human conditions make it impossible to always reach the most appropriate diagnosis or to predict with certainty a particular response to treatment.

Therefore, it should be recognized that adherence to these guidelines will not ensure an accurate diagnosis or a successful outcome. All that should be expected is that the practitioner will follow a reasonable course of action based on current knowledge, available resources, and the needs of the patient to deliver effective and safe medical care. The sole purpose of these guidelines is to assist practitioners in achieving this objective.

## Purpose and scope

The purpose of this guideline is to provide comprehensive information on best practices for robust radiomics analyses, including study design, quality assurance, data collection, impact of acquisition and reconstruction, detection and segmentation, feature standardization and implementation, as well as appropriate modeling schemes and evaluations. Interpretation of results along with possible pitfalls is also covered. At the end of the guideline, an outlook for future perspectives is provided. Radiomics is a very quickly evolving field of research. The present guideline will thus focus on established findings as well as recommendations based on the state of the art. This guideline recognizes hand-crafted and deep radiomics frameworks, though it primarily focuses on the former, i.e., radiomics workflows involving handcrafted features because this field is more mature than deep radiomics. An update will be carried out in the future once more studies and results have contributed to some consensus regarding the use of deep learning methods in radiomics. Although most methodological recommendations in the present document are valid for most medical image modalities, we focus here on nuclear medicine, and specific recommendations when necessary are made for PET/CT, PET/MR, and quantitative SPECT.

## Introduction

### Origins and evolution of radiomics

The notion of relating imaging information to prognostic and diagnostic clinical endpoints traces its origins to applications of computer pattern recognition in the 1960s, but its systematic application to quantitative imaging analysis dates to the beginning of the 1980s in areas such as computer-aided decision or diagnosis (CAD) [1]. Interest in this area was further spurred by the need to meet personalized medicine requirements analogous to the success of genomics in biological sciences at the turn of the millennium. Some early examples (not using the term “radiomics” at the time) include the investigation of correlations between ultrasound signal and breast tissue malignancy [2], CT imaging phenotypes with gene expression [3, 4], and between PET-based features and radiotherapy response [5].

**Fig. 1:**
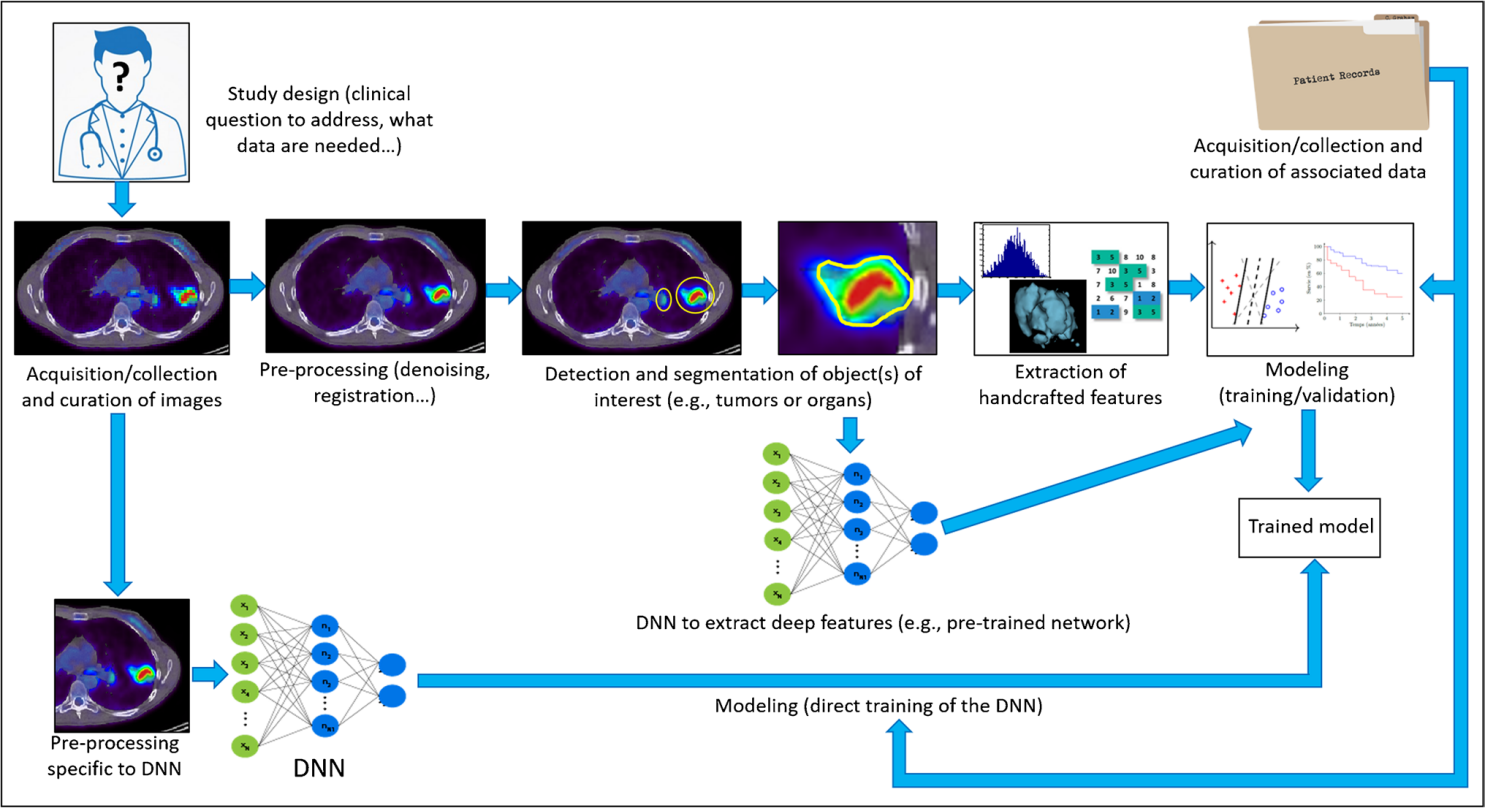
Top part illustrates the typical standard radiomics workflow, whereas the bottom part illustrates two different (among a myriad of possibilities) use of deep neural networks: direct training of a network using the input images or using a pre-trained network for extracting additional/alternative features from segmented tumor

Radiomics as a term was introduced in 2010 [6] and later formalized as a workflow based on machine learning in 2012 to denote the high throughput extraction of numerous quantitative metrics (including shape, intensity, filter-based, or textural features) [7] (Fig. 1). At the time, the scope was limited to radiological images (more specifically computed tomography (CT) in the field of radiotherapy applications). The aim was to provide a full macroscopic phenotyping of tumors that could reflect at least in part the underlying pathophysiological processes (such as necrosis, proliferation, etc.), down to the transcriptomic and genomic levels. The idea to extract features that could be computed by applying mathematical operations to the images (also referred to as engineered or handcrafted features) is not recent as stated above. Specifically, the use of such features in pattern recognition has existed for decades; e.g., Haralick texture analysis (subsequently becoming a very popular category of radiomics features) was introduced in 1973 [8] and quickly applied to computer-vision problems. Engineered features were also utilized as early as 1976 [9] in pathology (cytology) applications (and later introduced in commercial products in the 1980s). In the 1990s, engineered features were applied various modalities, including ultrasound [2], MR or SPECT to images of, as part of CAD systems [4, 10–12], while for positron emission tomography (PET) studies began to appear in 2003 [13], 2009 [5], and 2010 [14]. Radiomics is obviously not restricted to CT images of tumors and can be applied to images from other modalities in order to characterize both normal tissue and abnormal regions. As a field, it has seen an exponential growth (< 10 publications used the “radiomics” keyword in 2014, ~ 250 in 2018, and almost 2000 in 2020^1^). The vast majority of these studies investigated the characterization of tumors in CT, PET, and MR images with the usual clinical goals of improving or facilitating diagnosis (“digital biopsy”), discovering correlation with biological and genomics markers (radiogenomics), identifying aggressive or resistant tumor profiles, and predicting outcome (response to therapy, survival).

We can demarcate 4 eras for CAD relying on medical images, before and after the rise of radiomics. The period before the term was taken up in the seminal paper of P. Lambin in 2012 [7] could be called the “pre-radiomics” era. Studies during that period mostly design/extract a limited number of features from images in small cohorts of patients, and rely on basic statistical analysis to evaluate the differentiating/predictive power of these features. The era between 2012 and 2015 could be referred to as the “rise of radiomics” era. Following the seminal paper of 2012 and the highly cited work by Aerts et al. published in Nat. Communications [15] in 2014 (despite its flaws that were later emphasized [16]), the term “radiomics” was quickly adopted and increasingly used in publications. Studies started relying on a similar (although clearly not standardized yet) and larger set of handcrafted features, mostly intensity, shape, and textural features, still relying however on mostly basic statistical modeling in relatively modest cohort sizes. Starting around 2015, two eras evolved in parallel and continue to do so today. The first one could be called the era of “standardized radiomics.” It mostly consisted of studies aiming to improve practices and standardization of workflow and methods, driven by the development and success of the image biomarker standardization initiative (IBSI) that helped standardizing/standardize radiomic features nomenclature and implementation [17]. Cohorts of patients tended to increase in size, and the modeling step relied more and more on appropriate machine learning (ML) methodology. Most studies follow an established workflow, which consists in extracting handcrafted/engineered intensity, shape, and textural features from delineated volumes of interest (VOI). Starting around the same date [18], another era started: the era of “deep radiomics.” There, studies started/began to rely on deep neural networks to automate the detection/segmentation step, to extract alternative features, to directly model the endpoint with respect to the input image (with or without segmentation), or all of this simultaneously. Today, there are thus mainly three types of radiomics studies being published: first, the studies that still rely on the standard workflow, by extracting IBSI-compliant standardized handcrafted features, that are then used in the modeling step. Most of these studies rely on a ML pipeline for the modeling step. Second, the studies that use a deep learning (DL) approach for the entire analysis, without relying at all on the IBSI and the usual radiomics workflow [19]. Third, studies that address the task at hand with a combination of standard radiomics and DL. In these studies, DL is used only to improve, facilitate, or automate a specific part of the workflow, such as the detection and segmentation step or the extraction of alternative/additional features such as “deep” features (e.g., using pre-trained networks).

Any clinical application relying at least in part on imaging could potentially benefit from the development of radiomics. This includes prevention and screening, diagnosis, staging, prognosis, response to therapy, as well as radiotherapy planning guidance [20]. Radiomics has shown promising results in identifying tumor subtypes, aggressiveness as well as in predicting response to therapy and outcome of patients in several cancers, although most of these results have been obtained in small, retrospective, and monocentric cohorts [21, 22]. Reaching a higher level of evidence regarding the clinical value of radiomics requires carrying out studies of higher design quality and rigor, analyzing large (potentially prospective) multicentric cohorts of patients. Additionally, even the single-center studies have often had multiple limitations. Firstly, there are issues related to the image-degrading effects in PET, such as noise and partial volume effects, which can adversely impact the reliability of features quantified from PET images [23]. Further, computation of radiomic features may be affected by multiple factors such as variability in the acquisition and reconstruction parameters, the process to segment the tumors, and protocols to compute features [14, 24–27]. Following this, even in the computed features, studies have observed strong correlations, calling into question about whether these features are complementary [16, 28–31]. Another question has been that of the required number of patient samples, as evaluation of a large number of radiomic features with a small patient dataset can easily lead to false discovery [32, 33]. Furthermore, comparison of published PET radiomics studies is confounded by the variability in the definitions and protocols used to compute radiomic features [17]. Finally, there is a lack of consensus on the radiomic features that must be extracted, and the validation methodologies [32, 34]. Recent reviews of PET radiomic studies concluded that while PET radiomics is a promising field, very few papers perform in-depth validations, and the number of patient samples in most studies is insufficient [35, 36]. These reviews also emphasized on the role of standardization in the near future. Guidelines and recommendations constitute a tool that can help in addressing these concerns and facilitating the efforts in PET radiomics to come to fruition, thereby avoiding false discovery and waste of efforts.

### Definitions and overall workflow

Radiomics can currently be defined as the high-throughput extraction of image features from medical images in order to build diagnostic (e.g., differentiating between malignant and benign tumors), predictive (e.g., identifying non-responders to a specific treatment), and prognostic (e.g., predicting recurrence-free survival) models [7]. One very appealing promise of radiomics is its potential to identify informative combinations of features or patterns that cannot necessarily be appreciated with the naked eye, even the expertly trained ones [37].

Over the last few years, ML methods have become a crucial tool in radiomics for building and validating multiparametric models [19, 20, 38, 39]. Such methods are necessitated by the number and diversity of features, as typically hundreds of radiomics features are computed for each region of interest in addition to clinical contextual data and omics data, such as transcriptomics and genomics. The rapid advancement of DL in medical imaging [40] has also led to evolution of radiomics workflows towards the use of techniques based on deep neural networks (DNN). These can be used to automate and improve parts of the radiomics workflow, especially the detection and segmentation step or the feature extraction process [21], but ultimately these techniques could replace entirely the usual analysis workflow illustrated in Fig. 1 by simply inputting images into one or several DNNs [41]. Consequently, we subdivide radiomics-based approaches to imaging into 3 broad categories [19]:

1. *Hand-crafted (or Explicit/Engineered) radiomics*: this refers to approaches that perform explicit extraction of pre-designed radiomic features from the images. This is commonly followed by (i) univariate analysis (e.g., how much does each feature predict a certain outcome), and/or (ii) multivariate analysis using regression or ML algorithms. Such algorithms are used to identify a subset of relevant, non redundant features in the extracted radiomics feature set, as well as additional variables from non-imaging data. The selected subset of features are then used to train a model (also called *radiomics signature*, i.e., a specific combination of radiomics and potentially non-radiomics variables). In addition, we note that radiomics analysis can be applied at the region/volume-of-interest (R/VOI) level, or at a finer scale; e.g., generating a “parametric” image of a given radiomic feature, where feature value at a given pixel/voxel is computed via analysis of a neighborhood of that pixel/voxel [42].
2. *Representation learning (RL)-based radiomics*: this refers to approaches that aim to automatically discover features and patterns inherent in the images, and forgo the use of hand-crafted features. Neural networks (NN) are commonly used for this purpose, but other approaches exist as well, e.g., dictionary learning [43]. Neural networks directly learn from the images, and potentially, from additional inputs (e.g., non-imaging data). Neural networks can be shallow (1 hidden layer), but far more commonly, they have multiple hidden layers, which has resulted in an explosion of applications utilizing such deep NNs (DNNs) in the field of DL.
3. *Hybrid radiomics*: this approach involves combining the above-mentioned two frameworks in a number of possible ways. Examples include utilizing DL to generate features (e.g., from an intermediate convolution layer in a DNN; or from the final fully connected layer) followed by application of ML to the extracted features, to arrive at a radiomics signature [44], or combining deep features extracted from pre-trained DNN and handcrafted features [45].

Radiomics, as a whole concept, addresses both the data collection, curation and imaging aspects, as well as the aspects more related to artificial intellgience (AI), such as machine and deep learning techniques that can be used for either characterization of the images’ content or for modeling, or both.

Frameworks 2 and 3 are also sometimes referred to under the umbrella term deep radiomics, given the prevalence of DL methods in representation learning, and the use of DL somewhere along the workflow.

Regardless of the specific approach, data is required to first train and then evaluate a radiomics model. The dataset used to create a model is referred to as a *training*, *development*, *discovery*, or *exploratory* dataset. To assess the model, additional data are used that were not used for training. These datasets in the radiomics literature are often referred interchangeably to as *test* or *validation* datasets. However, it is important to distinguish between these two terms. *Validation* datasets are used for optimizing the modeling process (e.g., hyperparameters of a model), whereas *test* datasets are completely set aside until the final evaluation of the model. In this context, an *external test dataset* is understood to refer to a dataset that is only used for final evaluation and is also distinct from the training and validation sets in the sense of being obtained in a different institution, using a different scanner, during a different time frame or even analyzed by different readers. Such “externality” of the test set should be described thoroughly. For instance, a testing set that differs from training only by its time frame likely demonstrates less generalizability than a testing set from another institution with additional variability factors.

### Goals and scope of these guidelines

Goals: these guidelines aim at providing researchers and clinicians an updated and state-of-the-art relevant guide of good practices and recommendations for conducting radiomics studies in nuclear medicine imaging. Although the radiomics field is a quickly evolving field, the present document will try focusing on established recommendations and demonstrated pitfalls that should (and can) be avoided by researchers in the field or clinicians that would like to explore the transferability of radiomics in clinical practice. By relying on the present document, researchers and clinicians will contribute in improving the overall quality and reproducibility of radiomics investigations.

Scope: The present guidelines are dedicated to nuclear medicine imaging applications so most references and examples are related to these. Readers should nonetheless keep in mind that most of the methodological aspects addressed here are very often relevant to other image modalities as well, as they are quite independent on what image type is being exploited. The following aspects will be addressed: study design, data curation, image pre-processing, tumor detection and segmentation, features calculation, and modeling. In addition, the present guidelines focus on the “standard” radiomics workflow, as established in the beginning of the 2010’s, before methods based on DNN gained traction. Radiomics relying on DL techniques is developing quickly, but “deep” radiomics are less mature, and it would be quite difficult to provide a full set of guidelines and recommendations regarding specificities of the required amount of data, preferred architectures, and training procedures, etc. This is nonetheless addressed in the present document, especially as potential solutions for specific issues and challenges.

## Recommendations for radiomics in nuclear medicine

Quantitative image analysis has deep roots in the usage of nuclear medicine and especially positron emission tomography (PET) in clinical and research settings to address a wide variety of diseases. It has been extensively employed to assess molecular and physiological biomarkers in vivo in healthy and disease states, in oncology, cardiology, neurology, and psychiatry. Quantitative PET allows relating the time-varying activity concentration in tissues/organs of interest and the basic functional parameters governing the biological processes being studied. First radiomic studies in nuclear medicine have focused on PET, however given that SPECT images are now also quantitatively reliable [46], applying radiomics to SPECT is also feasible and relevant [45]. Radiomics can of course be applied to PET/MR similarly as in PET/CT, at least as far as the PET component is concerned.

This section is divided into subsections that follow the usual sequential steps in the workflow of radiomics studies, namely study design (2.1), imaging and data collection (2.2), image pre-processing (2.3), detection and segmentation of regions of interest (2.4), computing handcrafted features (2.5), and training and evaluation of models (2.6). To facilitate the use of this guideline, the actual recommendations made by the authors are highlighted in specific sections named “recommendations.”

### Study design

Overall, the majority of existing radiomics studies have moderate to poor quality, leading to non generalizable results and relatively low levels of evidence [47]. In order to improve over this current state of the art and avoid potential pitfalls, researchers and clinicians willing to carry out a radiomic study are encouraged to ensure they follow the most rigorous study design and quality assurance.

First, the clinical question to address must be clinically relevant and fully identified before the next steps can be considered. Secondary goals and other aspects of the study can be adapted and modified along the following steps, for example depending on the amount, details, and/or quality of data that are subsequently identified as available for analysis.

After identifying a clinically relevant question, the requirements to adequately answer the question must be listed and defined properly. These requirements are, for example, the number of patients required, performance of the current standard (clinical or other), type of image modality, the data analysis strategy, etc. Pre-registration of the requirements and the analysis plan may moreover reduce the chance of optimistically biasing findings [48].

There are already some recommendations published that can help properly design, auto-evaluate, and carry out a radiomics study.

#### Recommendations

Radiomics as a whole concept heavily relies on tools of AI applied to imaging, so position papers on AI for nuclear medicine are obviously relevant and are complementary to this guideline [49–53], as are some well-written reviews and position papers specifically on radiomics [21, 54–58].

For example, issues such as a discriminative bias due to data selection (race, gender, ethnicity...) are very important for “fairness” of developed models. Thus, potential sources of bias should be identified during data selection and be further investigated during the analysis. We recommend the involvement of an expert (bio)statistician to estimate an appropriate sample size for a given study. As mentioned elsewhere, e.g. [59], appropriate sample size strongly depends on the signal present in the images. The following “rule of thumb” can be followed: If the problem can easily be solved by an expert human observer, less than 100 images may suffice. If an expert human observer would really struggle to solve the issue, more than several hundreds of images are likely to be required, and excellent results may be difficult to achieve. Requirements for intermediate difficulty tasks fall somewhere in between. As a general rule, more and more diverse data are almost always better.

Other general recommendations such as the TRIPOD (Transparent reporting of a multivariable prediction model for individual prognosis or diagnosis) guidelines [60] or QUADAS (Quality Assessment of Diagnostic Accuracy Studies)-2 [61] that addresses patient selection, index test, reference standard, and flow and timing can be relevant for radiomics studies developing prognostic models. Other more specific ones should be relied upon [57, 62], such as for instance the IBSI workflow, nomenclature, implementation and reporting standards [17], and the radiomics quality scoring system, which can be used for auto-evaluation and relied upon to identify important points to address when designing studies [47, 59], although it should not be taken as an assessment of the correctness of results interpretation (see the example of Aerts et al. [16]). A very recent work provides a checklist and recommendations that can be followed for carrying out a rigorous radiomics study [63]. The recent “*best practices for algorithm development*” [49] and “*best practices for evaluation (the RELIANCE guidelines*)” [52] published by the SNMMI AI task force also largely apply to radiomics and are therefore highly relevant and complementary to the present guidelines. Finally, in order to optimize the impact on the research and clinical community, efforts should be made during the design of the study to promote and adhere to FAIR (Findable, Accessible, Interoperable, and Reusable data) principles [64], in order to increase availability of data and models and facilitate their sharing.

Beyond these general considerations, a reliable study design for radiomics consists in being well aware of all potential pitfalls associated with each step of the workflow for the envisioned analysis. Therefore, a proper study design should be prepared by accounting for all potential pitfalls and addressing them by their associated solutions or recommendations listed in the following guidelines in Sect. 2.2 to 2.6.

### Data acquisition, collection, and curation

The current guideline presents a multidisciplinary approach resulting from a group of professionals that are involved in preparing and extracting quantitative features from nuclear medicine images. Nuclear medicine technologists (NMTs) and nuclear medicine physicians are health professionals responsible for undertaking a range of nuclear medicine diagnostic and therapeutic procedures [65]. It is therefore the responsibility of the NMTs to ensure that the conditions prior, during, and after imaging will be compatible with the present guidelines, especially within the context of a prospective collection of images for radiomics investigations.

On the one hand, while preparing the acquisition and reconstruction protocols, it is fundamental that the medical physicist, NMT, and reporting physician communicate their specific needs. These needs should especially take into consideration that modern high-end PET/CT scanners may experience some image degradation, in order to comply with harmonization needs such as EARL [66, 67], which might require additional reconstructions for different use of the same acquired raw data. The NMT should critically carry out all acquisition and reconstruction protocols, being informed of their application. This integrative approach will guarantee the generation of high quality data for radiomics analysis, particularly if there are artifacts or irregularities in the process of image acquisition or reconstruction. For a number of diagnostic procedures, there is a strong component of patient preparation that aims at reducing tracer uptake in normal tissue while maintaining an optimal uptake in target structures [68]. The NMT is responsible for carrying out and documenting patient preparation. If it is not possible to carry out the recommended patient preparation procedure, feature extraction may be rendered invalid, or at best will need a specific training to identify images with artifacts.

One recent study evaluated the performance of existing EARL harmonization guidelines for PET/CT imaging to reduce the variability of radiomic features across different scanner models and reconstruction settings [66], with a 3D printed phantom scanned on different systems. Although EARL1 and 2 increased the number of comparable features compared to original clinical reconstruction in each center, a large percentage of radiomic features still exhibited significant differences even after harmonization, suggesting that, although useful, it may be insufficient to make all radiomic features usable in such a setting.

Finally, it should be emphasized on that although it has been suggested that alternative reconstruction settings compared to the standard clinical ones, which have been a priori optimized for visual analysis and detection rather than finer radiomics characterization, could perhaps lead to more discriminative features [25, 34], recent results suggest that it may not always be the case [69].

#### Recommendations

We cannot currently recommend optimized clinical acquisition/reconstruction settings specifically for the purpose of radiomics studies. We thus recommend to rely on current harmonization guidelines for PET/CT imaging that have been developed to make PET imaging as reliable and reproducible as possible across centers, as this for sure can improve robustness and reproducibility of the derived radiomic features [66]. Future harmonization guidelines are expected to more closely consider radiomics applications and build upon other recent investigations suggesting acquisition parameters and settings to minimize the variability of resulting radiomic features [25].

For radiomics analyses drawing on retrospective data images are typically already reconstructed and raw data are no longer available. Direct harmonization of reconstruction protocols is therefore usually not possible in the retrospective setting. It is well established that variability of a number of acquisition and reconstruction factors (including but not limited to scanner model and/or generation, uptake time, scan duration, reconstruction algorithm and parameters, post-filtering settings) can influence usual radiomic features values in PET, as reported in several studies [14, 27, 66, 70], although the resulting impact on their clinical relevance and differentiation power may not necessarily be strongly impacted [69, 71]. However, these studies also highlighted the fact that the sensitivity of radiomic features can vary greatly, with some exhibiting higher robustness to various factors than others.

Different use cases can be considered and different associated recommendations can be made for dealing with heterogeneity in acquisition and reconstruction factors:

### Recommendations

#### Training a model for local use

If the goal is to build a model for internal future use, we recommend collecting data with similar acquisition and reconstruction settings, in line with the established (and future) local clinical acquisition and reconstruction protocols, in order to minimize the variability of radiomic features distributions. However, note that the developed model will likely be more difficult to validate in an external setting with different acquisition and/or reconstruction parameters. The developed model is therefore unlikely to be used by other centers or research groups, unless the features selected for building the model are highly robust to the changes in acquisition and/or reconstruction parameters. Additionally, the model will need to be updated (i.e., re-trained or harmonized in some way) if changes occur in the local setting such as replacement of the scanner model and evolution of the acquisition and reconstruction protocols to meet clinical needs. Additionally, such a model would likely not be able to be used beyond research studies.

#### Building a more generalizable model (i.e., heterogeneous imaging data)

A more ambitious goal is to build a model with potential for external use (and an actual wide translation to clinical routine); we recommend collecting data used for training, tuning (validation), and testing exhibiting variability in acquisition and reconstruction parameters.

Given the major impact of these factors on radiomic features, the main recommendation in that context is therefore not to exploit uncorrected raw features in case of data presenting variability in acquisitions/reconstructions. It has been shown that in such a setting, either spurious correlations could be found or others could be hidden [72].

As a consequence, taking care of the issue in some way or another should always be performed in order to investigate if results can be improved. Given this issue is relatively recent and an active field of research, we recommend that all studies exploiting heterogeneous datasets should systematically report their results without and with the chosen correction/normalization/harmonization procedure(s). Given this specific issue has been under thorough investigation for only a few years, the advantages, disadvantages, and caveats for these procedures have not been explored fully within the radiomics context. Hence, we cannot make a recommendation concerning the method to use for dealing with heterogeneous imaging data. A number of methods have been proposed [73, 74]. Such methods operate either in the image domain or in the features domain. In the image domain, methods such as processing images with standard interpolation and filtering tools or using DL techniques such as convolutional neural networks (CNN) [75] and image synthesis, e.g., generative adversarial networks [76, 77], have been published in order to harmonize images and thus resulting radiomic features. In the feature domain several approaches have been investigated, such as eliminating non-robust features [78], modifying the features definition and implementation [79–81], or processing the features values using statistical methods such as normalization [82] or batch effect removal [72]. It is likely more efficient to perform some kind of harmonization or correction, rather than eliminating the non-robust features beforehand, which could lead to a loss of potential clinically relevant information [83], as most features exhibit at least some sensitivity to these factors. Using a posteriori harmonization through statistical methods of already extracted features has the advantage of being easier and faster to use than harmonizing images prior to feature extraction [73]. Among these, ComBat [84] seems to provide an available, operational, and efficient way of addressing the issue [85], although it is not without limitations and should not be used as is when its underlying assumptions are not met [85]. One limitation is that sufficient data with highly similar acquisition and reconstruction parameters, forming a single batch, should be present to estimate transformation parameters. Moreover, batch normalization methods assume that differences between batches reflect differences in acquisition and reconstruction parameters, and not actual differences related to patient characteristics. This assumption should be checked to avoid removing clinically relevant differences in feature values. Note that ComBat allows modeling actual differences related to patient characteristics (through a covariate matrix) to preserve them [84]. Variations of Combat providing improved robustness, as well as harmonization of previously unseen data, have also been proposed [86, 87].

### Image pre-processing

Various pre-processing steps can be envisioned for nuclear medicine images, including denoising [88] and partial volume effects correction (PVC) methods that generate corrected images to be used in subsequent steps [89]. These usually aim at improving the signal-to-noise ratio (for denoising) and the spatial resolution and quantification accuracy of images (for PVC) beyond what the reconstruction algorithm initially produced. Numerous methods have been published over the years, more recently including methods based on DL demonstrating state-of-the-art performance.

#### Recommendations

Although the repeatability of radiomic features obviously improves with better noise properties [70], the available literature does not suggest a significant impact of PVC using standard PET metrics [90] and a fortiori does not currently provide evidence that pre-processing images with denoising and/or partial volume effects provides significant improvement for radiomics application. We currently cannot recommend that such pre-processing should be systematically applied. If investigators wish to include such pre-processing of images they should include in the study a comparison with/without pre-processing in order to demonstrate their potential benefit on the resulting model performance.

### Detection and segmentation

The standard radiomics workflow relies on the assumption that the object(s) of interest to characterize (e.g., an organ, a tumor, thus in the present context of nuclear medicine imaging, a specific radiotracer uptake) first needs to be detected and then delineated in the image, before features are calculated.

In this specific step of the workflow, the reliance on manual or semi-automatic tools combined with the lack of standards forms one additional factor limiting reproducibility and acceptability of radiomics in PET. Even if all the other steps of the workflow would be perfectively standardized and similar across all users, relying on different delineation algorithms and therefore exploiting different volumes of interest would still lead to major differences and prevent reproducibility of the results, as various studies have shown specifically for PET radiomic features, including for their resulting clinical value [70, 91–93]. Beyond the segmentation of a single volume of interest, a much more challenging task is the accurate and precise delineation of multiple lesions. Most semi- or fully automated techniques developed prior the use of CNN assumed the object of interest was first detected and placed in a volume of interest as a pre-processing step, most often relying on a user intervention. This is why the delineation step is often considered as the most time-consuming bottleneck of the radiomics workflow, especially in the case of multiple lesions.

A large number of methods have been developed to address fully automatic segmentation of volumes of interest in PET images [94]. Initial efforts heavily relied on very basic threshold-based methods, which then evolved towards adapting more modern image segmentation techniques. This has now culminated in the use of state-of-the-art DL-based techniques such as the U-Net CNN architecture [95, 96]. This approach won the first challenge on PET image segmentation organized with MICCAI [97]. Likewise, all participants in the recent HECKTOR segmentation challenge on delineating primary tumors in head and neck PET/CT images used a U-Net variant [98, 99].

#### Recommendations

Based on existing results and previously published recommendations, including the report by the AAPM Task group 211 [94] and the MICCAI PETseg challenge [97]. For the purpose of radiomics studies:

i) Methods favoring positive predictive value over sensitivity in the segmentation performance should be preferred, as including parts of the uptake on the borders is likely to introduce more bias in resulting measurements of features (especially mean uptake but also specific textural features) due to partial volume effects (if no compensation/correction was applied to the images first) [28, 94].
ii) Methods based on fixed thresholding (e.g., 40 or 50% of maximum SUV) have the advantage of being quite reproducible across multiple readers. However, they should not be used without strong expert adjustments or correction for the purpose of radiomic studies, as they have been demonstrated to perform poorly especially in heterogeneous lesions [94, 97].
iii) (Semi)automated methods, rather than manual delineation, should be relied upon. Ideally, a consensus of several methods could be considered for improved performance. If no automated algorithm is available and only manual delineation can be considered, then ideally a consensus of at least three delineations by experts should be obtained, for example using approaches such as simultaneous performance and performance level estimation (STAPLE) technique [100]. However, this would likely restrict the analysis to small datasets. Alternatively, if only manual delineation by a single observer is feasible for the entire study, proper study design should include an analysis on a subset of patients to investigate the potential impact of inter-user variability on the model’s performance. For example, a model trained on patients delineated by one expert could be applied to test patients for which the delineation was done by a different expert.
iv) The current state-of-the-art methods for achieving fully automated PET image detection and segmentation are almost all based on DNN such as the U-Net architecture [101, 102], which has been very successful in medical image segmentation tasks, including in PET/CT imaging [98, 99, 103]. As the learning process relies on pixels/voxels or patches (i.e., each voxel, rather than the entire image, carries a label), the amount of data (i.e., the number of patients) required for an efficient training can be relatively small (i.e., ~ 100 datasets, not thousands). DL-based methods integrating objects of interest detection and segmentation may facilitate the full automation of this step of the radiomics pipeline [95, 104], allowing for radiomic analyses of hundreds or thousands of patients datasets in a more convenient and less time-consuming fashion. One limitation is that a sufficiently reliable ground-truth needs to be available to train the algorithm in the first place. To address this issue, simulation-based strategies are being proposed, where realistically simulated PET images with known ground-truth tumor boundaries are used to train the network [105], leading to more accurate segmentation and to further reduction in required amount of training data, with training with even *N* = 30 patients yielding a Dice score of 0.7. The generalizability and performance of these algorithms are however questionable as they may fail in new, previously unseen cases. Fully automated segmentation should therefore always keep human experts in the loop for quality assurance.
  Given the large number of segmentation methods that are present, a common question is how to choose the segmentation method to measure the radiomic feature. Preferably, the segmentation method chosen should be such that it yields accurate, precise, repeatable, and reproducible model prediction. Thus, these criteria may be used to evaluate segmentation methods prior to their application [94]. More recently, a framework to evaluate PET segmentation methods based on the task of quantifying features was proposed [106]. Additionally, no-gold-standard evaluation strategies are being developed, which are showing promise in evaluating segmentation methods based on how reliably these methods compute the true quantitative value, without access to a gold standard [107]. However, the evaluation of segmentation methods for radiomic feature quantification remains an area of active and future research.

Finally, it should be remarked that the recommendations above assume the desired radiomic workflow involves extracting features from the tumor’s metabolically active volume, that is delineated as accurately as possible. However, recent studies have highlighted that although good models could be obtained by relying on features extracted from accurately delineated tumor volumes, similar (or in some cases even better) performing models could be obtained by extracting features from different volumes of interest, such as for example smaller volumes within the lesion (avoiding the impact of partial volume effects on the borders) [108] or larger volumes of interest containing the tumor as well as its surrounding tissues or organs [109].

### Features calculation

#### Available software/open code

Radiomics software processes medical images and computes features from the region of interest. The choice of software has been shown to affect feature values [110–113]. Important image processing steps and commonly used features were standardized by the IBSI [17]. The use of IBSI-compliant software reduces or mitigates the effect of software on feature values, as long as the same image processing and feature computation parameters are used [114–116].

Several open-source radiomics software packages are available [19], which differ in use (e.g., command line or graphical user interface), degree of compliance with the IBSI standard, and support for PET imaging (such as e.g., automated SUV conversion or auto-segmentation tools). We do not provide an exhaustive list (which would never be up to date); however, some of the more commonly used packages are pyradiomics [117], SERA [118], LIFEx [119], MITK phenotyping [120], and CERR [121]. Others are cited in [113, 116]. Commercial software for radiomics analysis is also becoming available. Users should ensure commercial developers follow IBSI standards. A potential solution is that users could use the commercial software to process the publicly available IBSI benchmark datasets in order to check they obtain compliant values for their features of interest. Developers should be forthcoming about how their methods were validated.

#### Recommendations

We recommend using an existing software package unless there is an interest in developing specific image analysis and feature-calculation algorithms not provided by the existing packages. The package used should always be tested for compliance with the IBSI standard prior to use, as there is currently no accreditation procedure for new software releases. However, in future, researchers may propose novel approaches to quantify radiomic features with the objective of characterizing these features even more accurately and precisely. In that case, it is important that these approaches be entered in the IBSI process prior to usage.

## Computing features

When computing features from a region of interest, resulting feature values depend on how images are processed, as well as on feature-specific parameters. In recent years, experience has been gained on how to process PET imaging and set reasonable parameters, which is reflected in the recommendations below:

### Recommendations

1. PET voxel values should be converted into SUV values prior to feature computation. An external tool may be required, depending on the software used. It is recommended to cross-reference the produced SUV values to clinically certified viewing software.
2. If physical voxel spacing, determined by in-plane resolution and the distance between subsequent slices, differs between measurements in a dataset, voxel intensities should be resampled to a grid with a common (ideally isotropic for textural features) voxel spacing [57]. Resampling should preferably be done with a higher-order interpolator, such as a cubic spline, to avoid smoothing texture in the image [122]. Note that downsampling, i.e., to a common voxel spacing that is larger than that in the original image, may lead to aliasing artifacts and may require application of a low-pass filter [57, 123] prior to resampling. At the moment there are no clear indications whether upsampling or downsampling schemes are preferable but maintaining consistent isotropic voxel spacing across different measurements and devices is important for reproducibility. Also note that post hoc harmonization of features using statistical methods (see Sect. 2.2) is a possible alternative solution to interpolating to isotropic voxel sizes.
3. Textural features should be computed in 3D, unless there is a clear reason not to, e.g., because voxels are non-isotropic as the distance between slices exceeds in-plane resolution considerably.
4. Textural features can be computed with either a fixed bin number (FBN) or fixed bin size (FBS) methods. It has been shown in PET that FBS yields features with lower correlation with the corresponding number of voxels involved in the calculation (i.e., tumor volume) than with FBN [124]. However, since FBS instead introduces spurious correlation with SUV [125], there is no consensus as to the superiority of one over the other in terms of modeling performance. For comparison purposes, it may be useful to systematically implement and report both.
5. For the FBN discretization method, the recommended number of bins should lie between 4 and 64 bins. A higher number of bins typically leads to very sparse and uninformative texture matrices [30]. For the FBS discretization method the lower bound should generally be placed at 0.0 SUV. The recommended bin size is problem dependent. Typical bin sizes lead to forming between 8 and 64 bins in the ROI. Exceedingly small bin sizes, e.g., 0.01 SUV, should be avoided as this again will lead to very sparse and uninformative texture matrices.
6. Typically, texture matrices should be computed with default parameters as listed in the IBSI reference document.^2^ Caution should be taken with respect to software that may not use the same default settings.

## Choosing which features to implement

The number of features to implement in a given study is a design choice driven by several factors, including the statistical analysis methodology and the sample size. The larger the number of features, the larger the feature space and the chance to “discover” a useful feature, if the modeling step appropriately takes care of the dimensionality curse issue (see modeling Sect. 2.6). On the other hand, reducing the set of features to non redundant, reliable, robust ones (which requires defining criteria relied upon for the selection) has the advantage of simplifying the modeling step, since the statistical analysis will explore a smaller set of features, with the added benefit to reduce the chance of accidently identifying noise as being relevant.

### Recommendations

Features can be selected based on a number of criteria, including redundancy (with each other as well as with established clinical factors or tumor volume for example) and reliability (e.g., test–retest reproducibility, robustness with respect to changes in acquisitions, and/or reconstruction settings, etc.) and/or overall ability to discriminate specific patterns, relying on phantom and simulations analyses (see supplemental material Sect. 1).

We recommend using an existing software package and relying first on IBSI-compliant and standardized radiomic features (i.e., most commonly used handcrafted features). Alternative metrics such as e.g., heterogeneity-shape statistical metrics [13, 126, 127], CoLlAGe (Co-occurrence of Local Anisotropic Gradient Orientations) [128], or Riesz-covariance texture [129] not currently included in the IBSI can be of course added. In that case, the appropriate references should be provided along with a description of the feature(s), and most importantly, they should be evaluated alongside IBSI-compliant ones. Any newly designed handcrafted feature should be well described and justified, checked for redundancy with the existing features, and fairly evaluated for its potential discriminative power or benefit for endpoint under investigation.

## Dealing with multiple lesion cases

Two recommendations in the context of managing patients with multiple lesions (as in the case of lymphomas or metastatic diseases) can be made:

### Recommendations

Whatever the strategy adopted (e.g., lesion-based or patient-based) or endpoint studied, data from the same patient should be contained to one set and not split across training, validation, and test sets.

For lesion-based endpoints, such as digital biopsies or response to therapy, each lesion can be considered an instance, as in the single lesion context.

However, based on the current literature, it is not entirely clear what strategy or approach is to be recommended in the case of multiple lesions per patient when patient-based endpoints, such as survival, are investigated. Learning from multiple lesions is likely easier to do using any DL-strategy, e.g., as a single image, multiple instance learning, etc., compared to relying on handcrafted features extracted from each lesion. For handcrafted features, the main issue to address is that data from multiple lesions needs to be aggregated in some way. There are several strategies that could be used [130], and it is currently unclear if any could be recommended: (i) Use features from the merged collection of lesions, i.e., as if they were a single object, (ii) Use features for each lesion and then aggregate these, e.g., by averaging, (iii) Use features for only one of the lesions, chosen based on some criterion, e.g., the largest or the one with the highest SUV.

### Modeling

Most of the papers during the 2010–2015 period were criticized for relying on basic, inappropriate statistical analyses that led to overfitting and overoptimistic claims. The main limitations of these studies lay in the use of univariate analysis only (as opposed to multivariate analysis), no corrections for false discovery, (very) small sample sizes (< 100 – 50), and lack of evaluation of the findings in data not used for discovery/training [32, 57]. Radiomic researchers thus switched to techniques based on ML algorithms in order to better rank and select features, as well as combine them into multiparametric models through classifiers [38]. This move also included relying on a more comprehensive methodological framework compared to basic univariate statistical correlations, such as a proper split of data between training (for building models), validation (for optimizing models), and testing (for actually evaluating their performance) [19, 39].

The main modeling methods in radiomics used regression methods (e.g., logistic regression) and more recently ML-based methods, including those based on DL.

#### Supervised, semi-supervised, or unsupervised techniques

ML can be defined as “*a field of study that gives computers the ability to learn without being explicitly programmed*” [131]. ML first relied on calculating handcrafted features in the raw data (through, e.g., computer vision methods) and using these features as inputs of an algorithm designed to learn a specific task. This process is denoted today shallow learning (SL). In contrast, DL is a type of ML relying on the use of artificial neural deep nets with representation learning [132].

Supervised learning denotes the use of ML algorithms that learn using labeled data, i.e., the training dataset is provided along with the true labels that should be predicted. Unsupervised learning denotes the use of ML algorithms that learn with no labels being provided, which means the algorithm has to infer patterns from the data. Semi-supervised learning denotes ML training relying on part of the training data being labeled and the rest without label. In DL, semi-supervised training consists in iteratively updating the network parameters and the labels of the unlabeled data. If the algorithm learns to map inputs into optimized actions, this is denoted as reinforcement learning, i.e., goal-oriented tasks. These algorithms currently represent the main categories of ML, with supervised learning being the most common type in radiological sciences with applications ranging from detection, to diagnosis, to therapeutic interventions. However, several techniques are emerging to relieve the burden and cost of data labeling in supervised learning including: the semi-supervised approach mentioned above, transfer learning (using knowledge from other domains, such as natural images when learning medical ones), active learning (an interactive approach with human being involved), and more recently weak supervised learning, where the labels are assumed to be imprecise or noisy. Unsupervised learning is typically used for clustering or data reduction tasks while reinforcement learning is applied for optimizing sequential decision-making processes, in clinical management for instance [132].

#### Data leakage issue (training/validation/testing)

Learning algorithms are susceptible to overfitting. Therefore, a model’s performance should be evaluated in data that are different from the data used to build the model. Typically, the model architecture or hyperparameters need to be optimized and compared. In that case, the dataset designated for training should be used for this optimization using strategies such as K-fold cross-validation, leave-one-out cross-validation, or keeping a portion of the dataset exclusively for validation. Only after the model’s parameters have been fixed should the model’s performance be evaluated on the testing data set. Careful attention must be paid to prevent data leakage, which occurs when information from the testing data set is directly or indirectly shared with the model during training.

The training (sometimes called discovery, exploratory, or development) dataset is used to discover correlations and patterns between variables (among radiomic features themselves or between radiomic features and clinical factors) or between radiomic features and the chosen endpoint. An example of this would be to discover in a cohort that the tumor SUV_max_ is correlated with the response to chemoradiotherapy status (e.g., non-responders exhibit statistically higher SUV_max_ than responders). The validation dataset is then used to select and optimize some parameters of the trained model. In the same example, it would consist in identifying a threshold of SUV_max_ values leading to the best result for a specific criterion (e.g., accuracy to classify patients as non-responders). Finally, the performance of a finalized model is evaluated using the testing (also called evaluation) dataset, which contains only samples never seen in the training/validation phase(s). In the example, this would consist in applying the previously optimized SUV_max_ threshold to new patients and reporting the accuracy with which the patients are classified as non-responders.

#### Recommendations

It is therefore recommended to always train and evaluate (test) the developed models on different datasets or subsets of the available data. There are different rule of thumbs to split an available dataset into training and testing, such as 50% for training, 20% for validation, and the remaining 30% for actual testing, but these values can be changed. It could for instance be relevant to train a model on retrospectively recruited patients and then evaluate it on prospectively recruited patients. In case of multicentric cohorts, data from one or more centers can be used for training and validation, with data from remaining centers set aside for testing. It is important to ensure that instances (patients) from the training set are not leaked to the evaluation stage. It is better to have a validation set to optimize model parameters, although cross-validation in the training dataset can be a surrogate solution if there are not enough patients to split into training and validation sets.

For splitting the data, it is recommended to rely on stratified sampling when using a single split (note that a single split is inherently a limitation as performance may strongly depend on the split), whereas random splitting can be relied upon if numerous splits are performed and measurements are then averaged over the splits.

#### Data imbalance

Data imbalance occurs when one or more classes is substantially underrepresented or overrepresented in the training data set. Severe imbalance can hinder a model from learning meaningful relationships for minority or majority classes, whether or not the class distributions are representative of the overall population. It is often the case with radiomics studies as the clinically relevant task is usually to identify a small subgroup of patients (e.g., the 20% of non-responding patients, or the 10% of patients with very aggressive tumor subtypes). A common technique to address data imbalance is a 2-stage approach in which sampling is used to create equally distributed data sets for the first stage of model training, followed by fine tuning of the model using the full data set. Alternative solutions include selecting objective functions more robust to class imbalance, such as class weighting. A popular technique to balance datasets for training models is SMOTE (Synthetic Minority Oversampling Technique (SMOTE) [133] or its variant SMOTE-EEN (SMOTE followed by Edited Nearest Neighbour) [134], which create synthetic additional samples by linear combinations of existing ones in order for the minority class to be balanced. Recent radiomic studies including comparisons with and without the use of such techniques suggest they can improve the predictive modeling [135, 136]. Alternative new methods have been utilizing generative adversarial neural networks (GAN) methods for synthetic data generation and imbalance correction [137].

#### Recommendations

Although it is currently difficult to recommend one specific approach, it can be recommended to implement at least one of the existing techniques to facilitate the training of the models, especially in cases of extremely unbalanced data. Balancing of the data should be applied only to training data, not validation or test sets.

#### Feature selection and classifiers

One of the first studies to systematically compare techniques for feature selection combined with classifiers for radiomics application was published in 2015 and included 14 feature selection methods and 12 classifiers. The context was outcome classification of lung cancer patients using radiomic features from CT images [38]. The study showed that even proper ML methodology may not be sufficient to get robust results and solve issues associated with improper statistical analysis, as indeed the choice of the feature selection and classifier combination led to significantly different performance, with area under the curve (AUC) values ranging from 0.50 to 0.69. Although this particular study lacked proper hyperparameter selection (e.g., the size of the radiomics signature was set to 30 features, a relatively large number), later studies reported similar variations of results depending on the choice of the methods for modeling [138, 139].

#### Recommendations

Based on the current literature, it is difficult to recommend one ML pipeline over another, as the dependence on the application and the data of interest remains high [138, 140]. One clear recommendation that can be made is to not rely on a single method for feature selection and model building, but rather to implement and test several ones.

These observations also prompted the suggestion that implementing several different techniques and then generating a consensus could improve the prediction performance [141] (see below).

#### Ensemble/fusion

It is well known in ML that the combined use of several, different models (called ensembling), can lead to better performance [142]. Some popular learners use ensembling internally. For example, in random forests numerous trees are trained and then combined for the final prediction. Given that several different ML pipelines (using different methods for selecting features and combining them into multiparametric models) were shown to provide different levels of performance in several comparison studies [38, 138, 139], it is not unreasonable to hypothesize that these different models reach different predictions for the same patients. Thus, as human experts, reaching a consensus among them could lead to an overall better prediction. An example of this was shown in the context of breast lesion classification (as malignant or benign) in three different image modalities (full field digital mammography, DCE MRI, and ultrasound), where a standard radiomic approach and a DL-based analysis of the images were compared and fused; the fusion always produced the best classification results [143]. More recently, it was shown in the context of prognosis modeling for non-small cell lung cancer patients using radiomic features from FDG PET/CT images that averaging the output probabilities of three different modeling strategies (random forest, support vector machine, and logistic regression) could improve the resulting predictive performance [141].

#### Recommendations

Although the literature is relatively scarce at the moment, especially for NM applications, we can recommend to at least try implementing a simple consensus of different models through, e.g., majority voting or averaging of output probabilities when several pipelines have been implemented (which we recommend to do, see Sect. 2.6.5) and report if it improves the performance of the models.

#### Evaluating and interpreting models

The result of a radiomics analysis is a statistical model or an ensemble thereof. Such models need to be explainable and interpretable [58, 144, 145]:

- Transparency: why does the model yield a particular prediction?
- Justification: why is the yielded prediction acceptable?
- Informativeness: what new information does the model provide to clinicians?
- Uncertainty estimation: how reliable are the predictions?

Answering these questions requires characterizing the model itself and the influence of features in the model. Below are several characteristics of the model that should be assessed as thoroughly as possible when developing and evaluating a model:

#### Recommendations

- **Model performance**: Model performance is measured to assess how good a model is able to predict the endpoint of interest by comparing the predicted values with expected values. The following should be taken into account:
  - As explained in Sect. 2.6.2, performance of a model should be assessed using data that were not used to train the model [146].
  - One or several complementary metrics can be used to evaluate the performance of the models. For classification problems (e.g., benign vs. malignant or responders vs. non-responders), we recommend the use of area under the ROC curve (AUC), sensitivity, specificity, and accuracy (balanced accuracy in case of imbalance in the data to avoid a bias in the evaluation). The Matthews correlation coefficient (MCC) [147] is also a useful and recommended metric that takes into account all 4 types of classification results (false and true positives as well as false and true negatives) into one single score ranging from − 1 to + 1 which is quite easy to interpret [148]. For time-to-event analysis (e.g., prognostic models), the C-index [149] is a recognized metric assessing the fitness of the model score output with respect to true outcome. For relatively rarely encountered regression problems, where a numeric value is to be predicted, the mean squared error (MSE), root-mean-square error (RMSE), or Brier score metrics are typically computed.
  - Performance of a radiomics model should be compared to the following other models or assessments, when appropriate:
    - A naive model that always predicts the majority class, the mean or median outcome value, or the average risk or survival probability, depending on the type of endpoint. This provides a baseline value to compare model performance against.
    - A model based on (relevant) clinical parameters, including common image features such as tumor volume (or similar measurements related to the size of the uptake of interest), mean, or max SUV. This estimates the model performance that could be achieved by using only simple variables that can currently be obtained as part of standard analysis without the need for more complex radiomic analysis.
    - The assessment of clinical experts, such as that of one or more experienced radiologists, radiation oncologists, or nuclear medicine physicians, depending on the clinical context and application.
  - Performance of a radiomics model should be expressed using confidence intervals. In case a metric does not have an analytic expression for its confidence intervals, bootstrap confidence intervals may be used [150].
- **Model calibration**: Models that perform well are not necessarily well-calibrated. Well-calibrated models for categorical and survival endpoints respectively estimate class or survival probabilities that are similar to those observed in the data [151]. Thus, well-calibrated models can be used to estimate personalized probabilities for an endpoint. In addition to visual assessment of a calibration plot, we recommend to quantify calibration as follows:
  - Compute a linear fit on the calibration data. The intercept of the fit represents calibration-in-the-large and has an ideal value of 0. The slope of the fit is called the calibration slope and has an ideal value of 1.
  - Given sufficient samples (*n* ≥ 200 non-events and *n* ≥ 200 events), the calibration data may be fit using a non-linear fit (e.g., spline) to assess local deviations from ideal calibration.
  - A statistical test is sometimes used to assess calibration, i.e., the Hosmer–Lemeshow test for categorical endpoints [152] and the Nam-D’Agostino or Greenwood-Nam-D’Agostino test for survival endpoints [153, 154]. These statistical tests depend on the number of groups used to compute the test statistic and should therefore be used with caution [155].
- **Decision curve analysis**: For models that are aimed at offering decision support for clinical interventions, decision curves allow visualizing the benefit of using the model to guide the decision instead of offering the intervention to everyone or no one [156]. We recommend the use of a decision curve analysis for both categorical and survival endpoints [157, 158]. Clinical or other baseline models may likewise yield a decision curve with which the proposed model can be compared.
  Characterizing the response of the model to the underlying features is important for understanding why the model yields its predictions. It may also help identify whether a model incorporates relevant information, or has learned a spurious correlation [159, 160]. Below are several feature-based characteristics that should be assessed. Note that when features are implicit, e.g., when the model is a CNN, many of these characteristics cannot be directly assessed and other techniques developed specifically for these methods should be relied upon (see supplemental material) [58].
- **Variable importance**: Variable importance should be used to identify which features affect the predictions most. This can be done using model-specific or model-agnostic methods. For instance, variable importance can be determined from the variance–covariance matrix associated with linear models, or the depth at which a feature appears in a decision tree. Generic and model-agnostic approaches for assessing variable importance also exist:
  - Permutation variable importance: Permutation variable importance quantifies the reduction in model performance caused by permuting a feature [161]. More important features yield a greater reduction in model performance when permuted. Careful interpretation is required in case one or more features are not independent, e.g., because of correlation with another feature, and other, more direct approaches are recommended, such as explicitly removing features or conditional permutations [162].
  - Shapley values Shapley values [163] are the weighted averages of marginal contributions for each feature to the value predicted by the model for a single instance, e.g., patient. In other words, for each instance the Shapley value of a feature is the value this feature contributed to the predicted outcome value. Since the computational complexity grows exponentially with the number of features in a model, Shapley values are usually approximated [164]. Shapley values can be used to determine variable importance by computing the average absolute Shapley value for each feature over all instances in a dataset [165].
- **Feature attribution**: How features affect the predicted outcome should be evaluated as well. Again, this can be done using model-specific and model-agnostic methods. For example, in linear models, the model coefficient of each feature directly determines how a feature affects the outcome. Several generic, model-agnostic, methods exist as well [165]:
  - Partial dependence plot: A partial dependence plot (PDP) shows the estimated marginal effect of a feature on the predicted outcome [166]. Thus, a PDP can be created to show how the outcome depends on a feature value. A PDP is commonly computed by taking existing instances in the dataset, and averaging over predictions with an altered value of the feature of interest. As with permutation variable importance, care should be taken if the feature of interest is not independent, and when the model starts extrapolating [162].
  - Individual conditional expectation plot: Whereas a PDP only shows the average over instances, the individual conditional expectation (ICE) plot shows the underlying instances. ICE plots may help identify heterogeneous behavior [167]. The same caveats as with PDP apply.
  - Accumulated local effects plot: An accumulated local effects plot mitigates the extrapolation issue of partial dependence plots by computing and accumulating effects locally, i.e., using subsets of samples that share a similar value for the feature(s) of interest [168].
  - Shapley values: As explained above, Shapley values can be used to show how a feature contributes to the predicted outcome.
- **Instance-based explanation**: Instance-based explanation methods are used to explain how a model makes a decision for a single sample, i.e., instance.
  - Local proxy models: Local interpretable model-agnostic explanations (LIME) [169] and newer approaches such as Local rule-based explanations (LORE) [170] attempt to explain the predicted value of an instance by synthesizing data similar to the instance. Then, an interpretable model, such as a decision tree, is fit to the synthetic data. Such a model may then also explain if and why a feature is important locally [171].
  - Shapley values: As explained above, Shapley values can be used to show how a feature value contributes to the predicted outcome.

It is also recommended for researchers to collaborate with clinicials and end-users in the development of appropriate explainability methods before the model development.

#### Clinical relevance: impact on patients management

PET/CT radiomics may improve the patient management by enabling the non-invasive prediction of outcome or determination of cancer phenotypes. For example, FDG PET derived radiomic features could predict recurrence of cervical cancer [172]. Another example is the prediction of cancer recurrence in early-stage non-small cell lung cancer by FDG PET-CT radiomic features [173]. Prediction of cancer recurrence offers the potential to intensify treatment regimes, which might improve patient outcome. Despite the fact that these radiomics models were sometimes externally validated and therefore should be generalizable, such approaches are still not implemented in the clinical routine. It should be noted that such external validation is rarely done in an independent way by different teams. In addition, they are rarely validated in large cohorts.

PSMA PET-CT is gaining increasing importance for initial prostate cancer staging and has also been employed in radiomics analyses. For example, it was shown that PSMA PET-CT radiomics can distinguish between intermediate and high-risk prostate cancer [174]. Although this may have the potential to better select patients for tumor biopsies and treatment approach, such a methodology is not yet used in routine clinical practice for patient management.

#### Recommendations

This is why we recommend conducting radiomics studies with the highest possible rigor in all important steps described above, from study design and data collection and curation to the modeling steps including evaluation and explanation of the models. Indeed, quality insurance of each individual component of the radiomics pipeline is a good first step, but it will be beneficial to implement a general quality assurance system. Only by following as much as possible these guidelines will the community be able to establish and cross-validate performant, robust, generalizable, and interpretable radiomic models that may in the end find their way in clinical practice. Radiomic studies focusing on developing models with direct potential impact on patient management should include as much as possible an evaluation of this impact as part of the model performance evaluation.

Ideally, models developed with the highest rigor and then validated by independent teams in large multi-centric cohorts may have the potential to then drive clinical trials and studies where the result of these models could change/impact a patient’s treatment, finally demonstrating their clinical relevance and value. Additional value for the community and improved likelihood for clinical transfer could come from testing the algorithm in an actual department of nuclear medicine, providing a cost effectiveness analysis, a high level of automation, a publicly available algorithm, and availability of associated open data.

The Table 1 above summarizes the most important factors to properly conduct a radiomic study in nuclear medicine imaging.

**Table 1:** Summary of the steps for performing a radiomics analysis in nuclear medicine with their most important recommendations

Step	Most important recommendations
Study design	Properly define: i) the clinical context, ii) the endpoint of interest, iii) a reasonable dataset size required to carry out the study, given the potential data imbalance with respect to the endpoint, iv) the selection criteria and the process to collect and curate images and associated clinical information
Data collection and curation	Double check the quality and integrity of imaging data and their association with clinical information of patients. Record all imaging acquisition and reconstruction parameters for all patients in order to evaluate heterogeneity of these factors. Plan for appropriate management of this heterogeneity if it exists (e.g., implementing harmonization of images or of features)
Image pre-processing	If images are pre-processed beyond the standard reconstruction (e.g., filtering for denoising or applying partial volume effects correction), report results with and without these additional steps to evaluate the actual benefit on the resulting models
Detection and segmentation	Whatever the chosen volume of interest, ensure its determination is as accurate, robust, and reproducible as possible. Avoid fixed thresholding methods
Feature calculation	Follow IBSI recommendations especially for SUV conversion, voxel size interpolation, and intensity discretization prior to feature calculation. Use an existing software package and check it follows IBSI standards. Adhere to IBSI recommendations for features implementation, parametrization, and reporting. Justify properly which features are chosen and implemented
Modeling	Avoid information leakage and properly divide the available data in training/validation (with or without cross-validation) and testing sets. Justify properly the chosen modeling schemes (algorithms for feature selection, model building) and if possible implement several different ones. Investigate and report in depth the model performance (also in comparison to some baseline), calibration, and explainability, as well as evaluate the potential impact on patient management
Evaluation	End-to-end evaluation is required using internal hold-out test data or independent datasets (external testing), multi-centric validation being beneficial for evaluating the robustness and generalizability of the model

## Future perspectives

Over the last few years, the radiomics community has witnessed two important evolutions. The first one is the advent of a much more standardized context compared to the earlier years, mostly thanks to the efforts of the IBSI. It has thus become easier to understand and compare studies, or even to reproduce them.

The second one is the quick evolution of DL in all fields of imaging sciences, including of course NM and radiomics. As already discussed earlier, methods based on DL are more and more being considered to accelerate or facilitate some steps of the radiomic workflow or to solve specific issues such as for example harmonization of images as a pre-processing step. In addition, DL is being more and more considered as an altogether alternative solution to standard radiomics, by directly training DNN to predict specific endpoints from input images without going through all the usual steps involved in the classical radiomics approach (i.e., tumor detection and segmentation, extraction and selection of specific handcrafted features, then modeling). Although this raises other issues such as the requirement for large databases for efficiently training models or technical solutions to help networks learn with limited amounts of data, and the explainability and interpretability of the resulting models, this evolution is likely to continue in the near future. In spite of the theoretical considerations on the greater expressive power of “deep features” compared to “handcrafted features,” it has been shown that some handcrafted features are difficult to capture by CNNs of limited depth given limited training data [175]. Furthermore, CNNs can be negatively biased in capturing shape information [176] which can be important for a range of clinical tasks [17]. Consequently, handcrafted radiomic features may be complementary to deep features. The present guidelines will therefore need to be updated once “deep radiomics” become mature enough.

As we discussed above, a major concern in radiomics is the large number of candidate protocols, including different reconstruction, segmentation, and discretization procedures. Techniques based on objective task-based assessment of image quality have provided a mechanism to address these questions in medical imaging [106]. Thus, exploring the use of task-based assessment to quantify radiomic features is another exciting area of future research. One major challenge with such evaluation is the lack of ground-truth radiomic feature values. To address similar challenges in quantitative imaging, no-gold-standard evaluation techniques are being developed [107]. Extending these techniques to optimize radiomic feature quantification protocols may provide a mechanism to optimize these features. Another research frontier is delta radiomics, which involves computing longitudinal changes in radiomic features in response to therapy [177, 178]. These changes can then be used in making clinical decisions such as early prediction of therapy response.

In order to validate models in large, multi-centric cohorts, some harmonization can be beneficial [179], although this is still an open area of research, with several candidate approaches being investigated, including deep learning-based image synthesis [180].

Another important future development is expected to lie with the combination of radiomics with other -omics fields and types of data, including but not limited to histopathology, genomics or dosimetry, and toxicity data, which clearly raises other modeling and validation issues.

As radiomics evolves further understanding of these features and their relationship to underlying biology will be demanded, relying and expanding on existing studies [4, 181–184].

## Liability statement

This guideline summarizes the views of the EANM physics committee and SNMMI Physics, instrumentation, and data science committee. It reflects recommendations for which the EANM/SNMMI cannot be held responsible. The recommendations should be taken into context of good practice of nuclear medicine and do not substitute for national and international legal or regulatory provisions.

## Supplementary Information

Below is the link to the electronic supplementary material.

Supplementary file1 (DOCX 53 KB)

## Footnotes

1 Source Web of Science (searching in all databases, restricted to journal papers). Note these numbers thus do not incorporate any study before 2012. All studies using textural or other handcrafted features but not relying on the term “radiomics” are not included either in these numbers.

2 https://ibsi.readthedocs.io/en/latest/.
