# Supplementary material for: Joint EANM/SNMMI guideline on radiomics in nuclear medicine: Jointly supported by the EANM Physics Committee and the SNMMI Physics, Instrumentation and Data Sciences Council
Source: Eur J Nucl Med Mol Imaging. 2022 Nov 3;50(2):352–75. doi: 10.1007/s00259-022-06001-6 (PMC9816255; doi:10.1007/s00259-022-06001-6)
Supplement: Supplementary file 1 — Supplementary file1 (DOCX 53 KB) [file 259_2022_6001_MOESM1_ESM.docx]

# **Guideline on Radiomics in Nuclear Medicine**

**Supplemental** **material**

1. **Phantom Validation - Volume dependence, reproducibility, inter-scanner variability**

***Rationale:***

PET radiopharmaceutical shape, intensity and distribution patterns quantitatively measured as radiomic features may reflect the metabolic and pathological state of cancer and help predict patient response to a chosen therapy, as described in the introduction. The appropriate clinical use of any radiomic feature would however benefit from a comprehensive validation process. The reliability and reproducibility of PET radiomic features is still a matter of debate [1] and there is a growing need for standardization. Aiming for the goals mentioned above, several approaches have already reached successful results as exclusion of volume dependent features [2]. In the challenge to mimic the most realistic patient tumor texture in a reproducible way, some heterogeneity phantoms were introduced using simple syringe inserts [1], 3D printing approach [3] or computer navigated point source movements in the PET field of view [4] revealing very good reproducibility results. While there have been many approaches already for MRI and CT radiomics features, this section generally describes the validation of PET radiomics features. These recommendations are also relevant for SPECT.

In case of a study in a multi-center environment there is an inevitable and latent scanner effect resulting in non-identical image texture alterations between different scanners. This scanner effect originated from the different detector realization of the PET ring and the different reconstruction algorithms. By definition, a feature, which precisely reflects textural properties should be selective in value for scanners representing different image quality. Otherwise one may receive similar results from different scanners due to the insensitive nature of the feature to the alterations in texture due to the scanner effects. Harmonization efforts have been initiated in two different ways: as a post reconstruction process [5,6], or as an image quality harmonization prior to the calculation of features [3,7]. Reproducible and repeatable phantom solutions have the potential to answer methodological questions emerging in multi-scanner studies.

Hereby we identify the most fundamental methodological considerations necessary to investigate during the preparation for a successful PET radiomics study based on phantom validation. The rationale of phantom validation consists of the following criteria to be met: a reliable image derived tumor heterogeneity parameter (HeP) has to be volume independent, reproducible, and being able to express quantitatively the degree of heterogeneity.

***Measurements and evaluation:***

To fulfill the goal of validating reliable tumor heterogeneity parameters described in the rationale section above, two types of phantom measurements are recommended:

1. ***Uniform cylinder phantom:***

Although it can be questioned to evaluate the relevance of textural features as HeP in uniform phantoms, the purpose of this measurement is to evaluate the reliability of a given set of HeP proposed for PET investigations in terms of volume dependency.

A uniform cylinder with diameter of about 20 cm (17 to 22 cm) and length sufficient to cover the entire axial field of view (FOV) should be filled with ^18^F-FDG according to the EARL Harmonization Standard Calibration QC protocol [27–30]: 70 ± 5 MBq activity filled in the phantom at the start of the acquisition and 2 bed position scan performed with a minimum of 5 minutes scan duration per frame.

Due to the limited spatial resolution of the scanners the texture evaluation could provide relevant information only for lesions above a minimum volume of interest. In further HeP parameters highly correlated with the delineated volume have questionable information content in regards to the texture [1]. The acquired uniform cylinder PET data should be reconstructed with the routine clinical reconstruction method and settings defined by the standardized operation procedure (SOP) of the nuclear medicine department. Ten separate spherical volumes of interest (VOIs) should be placed on the reconstructed PET images with volumes ranging from 0.5 ml to 1000 ml. The intended HePs of use should be calculated on all of the different volume VOIs and their values should be plotted [2]. Since, all HePs include volume dependency for volumes approximately below 30 ml [2], the VOI results above 30 ml should be checked. If there is still a tendency of increasing, decreasing or random behavior of HePs with increased volumes, then the actual HeP should be considered to be neglected in the study.

1. ***Heterogeneous phantom:***

The purpose using heterogeneous phantom measurement is to mimic the uptake of a tumor and to validate HeP inter-scanner variability. The simplest way to create a heterogeneous phantom is called a revolver phantom insert ensemble from 7 pieces of syringes. This insert should be filled with ^18^F-FDG as follows: 2 syringes at the start of the acquisition with 20 kBq/ml, 3 syringes with 40 kBq/ml and 2 syringes with 80 kBq/ml [2]. This phantom preparation represents the typical activity concentration range in F18-FDG studies. The revolver insert could be placed in the National Electrical Manufacturers Association (NEMA) image-quality phantom (Data Spectrum Corporation, Hillsborough, NC) attached to its lung insert and the background volume of the phantom should be filled with 5 kBq/ml activity concentration ^18^F-FDG. PET acquisition of the phantom set should be performed in at least 2 bed positions each with a minimum of 5 minutes scan duration. This image acquisition should be repeated 4 times. Clinically preferred image reconstruction should be performed on the acquired PET raw data together with reconstructions required by the actual study (e.g. EARL standard 1.0 or 2.0, etc.). The high uptake volume imitating a lesion should be defined as voxels with SUV>2.5 times the background within a boundary box. Mean value and standard deviation shall be calculated for each heterogeneity parameter from results of 4 independent image sets. These calculations need to be carried out for each predefined reconstruction method. The ratio of the standard deviation and mean (Coefficient of Variation: CV) should be used as the measure of error for the reproducibility of the selected parameter. In case a certain HeP does not meet the criteria of CV less than 10%, this HeP should be considered to be excluded from the study. This simple phantom setup provides an easy and reproducible method to measure the reliability of HeP results on a single system or for the purpose of inter-scanner or multi center studies. In addition, more sophisticated and labor sensitive methods may be applied as well such as 3D printed anthropomorphic tumor phantom inserts [3] or robotic arm moved point source measurements [4] that mimic tumor activity uptake. However, the scope of this guideline is to provide simple and easy to reproduce solutions for good practice that do not necessitate any special equipment.

1. **Use of simulations**

Simulations provide the opportunity to study data acquisition, image reconstruction and image processing methods, under realistic conditions, having knowledge of reference truth. Different categories of computational phantoms, particularly mathematical, voxelized and boundary representation (BREP) phantoms, have been and can be used, including for virtual clinical trials that imitate clinical experiments [1]. In the particular context of radiomics, this enables study of accuracy and precision of different radiomics features, for different parameters (e.g. post-smoothing filters, delineation method, etc.). Elimination of radiomics features that are not robust against image acquisition and reconstruction settings has been suggested in the literature [2]. Physical phantoms can also be used for these purposes [3]; at the same time, physical phantoms are often not as realistic (in terms of patient anatomy, physiology, intra-tumoral heterogeneity, etc.), though there are ongoing efforts in this direction [1]. Given the significant improvements towards more realistic simulation studies, including (1) introduction of preclinical and clinical (anthropomorphic) models, coupled to (2) effective data simulation packages, simulation studies have gained significant potential to screen the numerous existing engineered features in the literature (and future emerging ones) [1]. For instance, there are a wide number of radiomics features that are highly sensitive to noise which can be identified in this way [4]. Subsequent to this, effective features may be evaluated in real studies, e.g. to assess repeatability (test-retest), reproducibility, and performance as biomarkers of disease [5] [6].

For a subset of applications, simulation studies can be utilized in the context of transfer learning for classification purposes [7]. As an example, in the diagnostic context, if disease can be properly simulated; e.g. tumor absent/present (2-class problem), or tumor absent/benign/malignant (3-class problem), simulations can be used to derive an initial radiomics models, to be taken up as starting-point for subsequent tuning/improvements to the models in real studies. Furthermore, as radiomics applications tend to involve segmentation, simulations can be utilized to develop initial segmentation models that can be further enhanced when applied to real studies [8]. Due to the complex and laborious task of manual segmentation, the relative scarceness of annotations are significant obstacles for deep learning models to be generalizable in clinical settings. In fact, there is increasing evidence [9, 10] that the success of DL methods depends on the quality of annotations (taken as reference truth) and due to the insufficient knowledge of the ground truth, it is found that annotations depict considerable intra-observer [11] and inter-operator variability [12] [13]. Besides, in radiation oncology sometimes the organs-at-risk are only roughly labeled [10]. These can mislead the training of DL models. Moreover, real images with low signal-to-noise ratios and heterogeneous tracer distributions are not usually used for evaluation of proposed segmentation techniques [14] and the commonly utilized augmentation methods in natural images for the training of deep models are not necessarily as valid for medical images [15].

Thus, a very important contribution to this end can come from simulations, where ground truth is known, instead of purely relying on manual delineations as ground truth. Unlike real patient data, the detailed anatomy of simulated phantoms provides the ground truth i.e. “gold standard” to be used for standardized protocol for quantitative evaluation of segmentation and analysis techniques. Based on the recommendations by the task group 211 of the AAPM (American Association of Physicists in Medicine), the thorough, consistent and sufficient evaluation of developed PET auto-segmentation algorithms should be done in three phases including the phantom image, a combination of physical phantoms images and numerically simulated phantoms images, and clinical images [14], which we similarly recommend.

1. **Regarding confidence intervals for performance of models**

Creating confidence intervals this way may require creating an ensemble of models, instead of a single model. For deep learning this may be computationally prohibitive. To resolve this issue, different implicit ensembling methods have been devised where models share some parameters, e.g. snapshot ensembles [8], and divergent ensembles [9]. If the model generates a posterior distribution for the outcome, such as Bayesian neural networks [10] such distributions can be sampled to determine confidence intervals as well.

1. **Methods for explainability and interpretability of deep neural networks**

- **Deep-learning specific methods**: Deep-learning algorithms can yield well-performing models, but worryingly, they may easily learn shortcuts that do not generalise well [11,12]. As such performance of deep learning algorithms should be assessed in independent datasets. Note that many of the methods for assessing transparency and justification are based on features. Features are often implicit in deep learning and do not offer a meaningful explanation. Instead, a deep-learning model is usually explained based on the input scan as a whole. Below are some of the approaches that have been identified so far [13]. As explainable deep-learning is an area of active research, newer approaches may have been discovered that are not included in these guidelines:
  - Occlusion: Important parts of a scan may be identified by occluding patches of the scan and determining the resulting response of the deep-learning model [14]. When important parts of the scan are occluded, model performance will noticeably. While this approach was originally defined by introducing completely non-informative patches for occlusion, more recent methods extend the general concept by filling in occluded patches with more realistic data. For example, Fong and Vevaldi [15] perturb patches through blurring, adding noise, etc., and Agarwal et al. used a generative model to fill out patches [16].
  - Saliency maps: Saliency maps are used to visualise the contributions of the neurons in a neural network to each point in the scan. Methods to create saliency maps aim to highlight important regions in the scan. Recent work indicates that many proposed methods are invariant to network randomisation, especially in top layers [17,18]. Such methods therefore do not offer a sound explanation. Other methods are sensitive and can be used, e.g. integrated gradients[19], SmoothGrad [20], GradCAM [21], and DeepLIFT [22] were able to show a sound explanation. An open research question is whether saliency maps are actually able to convey the reasoning of the neural network, and saliency maps should be interpreted with care.
  - Uncertainty maps: Bayesian neural networks have the ability to distinguish between aleatoric and epistemic uncertainties [10]. Epistemic uncertainties are due to uncertainties in model parameters and can typically be reduced by increasing the size of the training dataset. Epistemic uncertainty can be used to identify when a model starts to extrapolate beyond training data, and is therefore important for medical applications. Aleatoric uncertainties are induced by noise in the input image and can typically only be reduced by improving scan quality. The spatial transformation of the input images can also increase the uncertainty [23].
  - Spectral relevance analysis: Unlike the above approaches which are local and instance-based, spectral relevance analysis (SpRAy) is a global approach that aggregates saliency maps over different instances by spectral clustering [11] allows for assessing whether certain parts of a scan are consistently considered to be important. This may indicate if a neural network for example has learned text embedded in a scan, or learned to look at the center of a scan because all training data were centered on a lesion. Hence, SpRAy may be a useful tool to identify shortcut learning.
  - Concept activation vectors: Testing with concept activation vectors (TCAV) relies on the use of simple images that define a concept, e.g. striped patterns for a zebra, to understand if this concept activates a neural network [24]. TCAV has been extended to the use of morphological and texture features as concepts [25]. This may allow for assessing whether a neural network has learned typical features such as MATV and mean and max SUV.

References

1. Valladares A, Beyer T, Rausch I. Physical imaging phantoms for simulation of tumor heterogeneity in PET, CT, and MRI: An overview of existing designs. Med Phys. 2020;47:2023–37.

2. Forgacs A, Pall Jonsson H, Dahlbom M, Daver F, D DiFranco M, Opposits G, et al. A Study on the Basic Criteria for Selecting Heterogeneity Parameters of F18-FDG PET Images. PloS One. 2016;11:e0164113.

3. Pfaehler E, van Sluis J, Merema BBJ, van Ooijen P, Berendsen RCM, van Velden FHP, et al. Experimental Multicenter and Multivendor Evaluation of the Performance of PET Radiomic Features Using 3-Dimensionally Printed Phantom Inserts. J Nucl Med Off Publ Soc Nucl Med. 2020;61:469–76.

4. Forgacs A, Kallos-Balogh P, Nagy F, Krizsan AK, Garai I, Tron L, et al. Activity painting: PET images of freely defined activity distributions applying a novel phantom technique. PLOS ONE. Public Library of Science; 2019;14:e0207658.

5. Orlhac F, Boughdad S, Philippe C, Stalla-Bourdillon H, Nioche C, Champion L, et al. A Postreconstruction Harmonization Method for Multicenter Radiomic Studies in PET. J Nucl Med. Society of Nuclear Medicine; 2018;59:1321–8.

6. Da-ano R, Masson I, Lucia F, Doré M, Robin P, Alfieri J, et al. Performance comparison of modified ComBat for harmonization of radiomic features for multicenter studies. Sci Rep. Nature Publishing Group; 2020;10:10248.

7. Choe J, Lee SM, Do K-H, Lee G, Lee J-G, Lee SM, et al. Deep Learning-based Image Conversion of CT Reconstruction Kernels Improves Radiomics Reproducibility for Pulmonary Nodules or Masses. Radiology. 2019;292:365–73.

8. Huang G, Li Y, Pleiss G, Liu Z, Hopcroft JE, Weinberger KQ. Snapshot Ensembles: Train 1, get M for free. ArXiv170400109 Cs [Internet]. 2017 [cited 2021 Sep 21]; Available from: http://arxiv.org/abs/1704.00109

9. Osband I, Blundell C, Pritzel A, Van Roy B. Deep Exploration via Bootstrapped DQN. ArXiv160204621 Cs Stat [Internet]. 2016 [cited 2021 Sep 21]; Available from: http://arxiv.org/abs/1602.04621

10. Kendall A, Gal Y. What Uncertainties Do We Need in Bayesian Deep Learning for Computer Vision? ArXiv170304977 Cs [Internet]. 2017 [cited 2021 Sep 21]; Available from: http://arxiv.org/abs/1703.04977

11. Lapuschkin S, Wäldchen S, Binder A, Montavon G, Samek W, Müller K-R. Unmasking Clever Hans predictors and assessing what machines really learn. Nat Commun. 2019;10:1096.

12. Geirhos R, Jacobsen J-H, Michaelis C, Zemel R, Brendel W, Bethge M, et al. Shortcut learning in deep neural networks. Nat Mach Intell. 2020;2:665–73.

13. Gilpin LH, Bau D, Yuan BZ, Bajwa A, Specter M, Kagal L. Explaining Explanations: An Overview of Interpretability of Machine Learning. ArXiv180600069 Cs Stat [Internet]. 2019 [cited 2021 Sep 21]; Available from: http://arxiv.org/abs/1806.00069

14. Zeiler MD, Fergus R. Visualizing and Understanding Convolutional Networks. In: Fleet D, Pajdla T, Schiele B, Tuytelaars T, editors. Comput Vis – ECCV 2014. Cham: Springer International Publishing; 2014. p. 818–33.

15. Fong R, Vedaldi A. Interpretable Explanations of Black Boxes by Meaningful Perturbation. 2017 IEEE Int Conf Comput Vis ICCV. 2017;3449–57.

16. Agarwal C, Nguyen A. Explaining image classifiers by removing input features using generative models. ArXiv191004256 Cs Stat [Internet]. 2020 [cited 2021 Sep 21]; Available from: http://arxiv.org/abs/1910.04256

17. Adebayo J, Gilmer J, Muelly M, Goodfellow I, Hardt M, Kim B. Sanity Checks for Saliency Maps. ArXiv181003292 Cs Stat [Internet]. 2020 [cited 2021 Sep 21]; Available from: http://arxiv.org/abs/1810.03292

18. Sixt L, Granz M, Landgraf T. When Explanations Lie: Why Many Modified BP Attributions Fail. ArXiv191209818 Cs Stat [Internet]. 2020 [cited 2021 Sep 21]; Available from: http://arxiv.org/abs/1912.09818

19. Sundararajan M, Taly A, Yan Q. Axiomatic Attribution for Deep Networks. ArXiv170301365 Cs [Internet]. 2017 [cited 2021 Sep 21]; Available from: http://arxiv.org/abs/1703.01365

20. Smilkov D, Thorat N, Kim B, Viégas F, Wattenberg M. SmoothGrad: removing noise by adding noise. ArXiv170603825 Cs Stat [Internet]. 2017 [cited 2021 Sep 21]; Available from: http://arxiv.org/abs/1706.03825

21. Selvaraju RR, Cogswell M, Das A, Vedantam R, Parikh D, Batra D. Grad-CAM: Visual Explanations from Deep Networks via Gradient-based Localization. Int J Comput Vis. 2020;128:336–59.

22. Shrikumar A, Greenside P, Kundaje A. Learning Important Features Through Propagating Activation Differences. ArXiv170402685 Cs [Internet]. 2019 [cited 2021 Sep 21]; Available from: http://arxiv.org/abs/1704.02685

23. Wang G, Li W, Aertsen M, Deprest J, Ourselin S, Vercauteren T. Aleatoric uncertainty estimation with test-time augmentation for medical image segmentation with convolutional neural networks. Neurocomputing. 2019;335:34–45.

24. Kim B, Wattenberg M, Gilmer J, Cai C, Wexler J, Viegas F, et al. Interpretability Beyond Feature Attribution: Quantitative Testing with Concept Activation Vectors (TCAV). ArXiv171111279 Stat [Internet]. 2018 [cited 2021 Sep 21]; Available from: http://arxiv.org/abs/1711.11279

25. M. G, V. A, S. M-M, H. M. Concept attribution: Explaining CNN decisions to physicians. Comput Biol Med. 2020;123:103865.
